# Expressive Interviewing Agents to Support Health-Related Behavior Change: Randomized Controlled Study of COVID-19 Behaviors

**DOI:** 10.2196/40277

**Published:** 2023-08-01

**Authors:** Ian Stewart, Charles Welch, Lawrence An, Ken Resnicow, James Pennebaker, Rada Mihalcea

**Affiliations:** 1 School of Computer Science and Engineering University of Michigan Ann Arbor, MI United States; 2 Department of Mathematics and Computer Science University of Marburg Marburg Germany; 3 Center for Health Communications Research University of Michigan Ann Arbor, MI United States; 4 School of Public Health University of Michigan Ann Arbor, MI United States; 5 Department of Psychology University of Texas at Austin Austin, TX United States

**Keywords:** expressive writing, motivational interviewing, dialogue systems, counseling, behavior change, text analysis, COVID-19, mental health, automated writing, writing system, stress, psychological health

## Abstract

**Background:**

Expressive writing and motivational interviewing are well-known approaches to help patients cope with stressful life events. Although these methods are often applied by human counselors, it is less well understood if an automated artificial intelligence approach can benefit patients. Providing an automated method would help expose a wider range of people to the possible benefits of motivational interviewing, with lower cost and more adaptability to sudden events like the COVID-19 pandemic.

**Objective:**

This study presents an automated writing system and evaluates possible outcomes among participants with respect to behavior related to the COVID-19 pandemic.

**Methods:**

We developed a rule-based dialogue system for “Expressive Interviewing” to elicit writing from participants on the subject of how COVID-19 has impacted their lives. The system prompts participants to describe their life experiences and emotions and provides topic-specific prompts in response to participants’ use of topical keywords. In May 2021 and June 2021, we recruited participants (N=151) via Prolific to complete either the Expressive Interviewing task or a control task. We surveyed participants immediately before the intervention, immediately after the intervention, and again 2 weeks after the intervention. We measured participants’ self-reported stress, general mental health, COVID-19–related health behavior, and social behavior.

**Results:**

Participants generally wrote long responses during the task (53.3 words per response). In aggregate, task participants experienced a significant decrease in stress in the short term (~23% decrease, *P*<.001) and a slight difference in social activity compared with the control group (*P*=.03). No significant differences in short-term or long-term outcomes were detected between participant subgroups (eg, male versus female participants) except for some within-condition differences by ethnicity (eg, higher social activity among African American people participating in Expressive Interviewing vs participants of other ethnicities). For short-term effects, participants showed different outcomes based on their writing. Using more anxiety-related words was correlated with a greater short-term decrease in stress (*r*=–0.264, *P*<.001), and using more positive emotion words was correlated with a more meaningful experience (*r*=0.243, *P*=.001). As for long-term effects, writing with more lexical diversity was correlated with an increase in social activity (*r*=0.266, *P*<.001).

**Conclusions:**

Expressive Interviewing participants exhibited short-term, but not long-term, positive changes in mental health, and some linguistic metrics of writing style were correlated with positive change in behavior. Although there were no significant long-term effects observed, the positive short-term effects suggest that the Expressive Interviewing intervention could be used in cases in which a patient lacks access to traditional therapy and needs a short-term solution.

**Trial Registration:**

Clincaltrials.gov NCT05949840; https://www.clinicaltrials.gov/study/NCT05949840

## Introduction

The COVID-19 pandemic has had a range of adverse effects on people across the world, increasing stress and anxiety for many. As of February 2023, the United States had recorded over 100 million COVID-19 infections and 1.1 million deaths. Actions taken by individuals, such as getting vaccinated, getting tested, or wearing a mask, can reduce the spread of the disease. Reducing stress during the pandemic can have important psychosocial and behavioral outcomes, especially for people who feel a loss of control over their lives as a result of pandemic restrictions [1,2].

Expressive writing is a behavioral intervention paradigm in which people are encouraged to explore their emotions and thoughts about significant life events. This method has had a positive impact on participants’ physical and mental health [3,4], including a decrease in physician visits, adoption of positive behavior, and improved moods. Along similar lines, motivational interviewing is a counseling technique that leverages a person’s intrinsic motivation and values to help them change their behavior. Motivational interviewing is known to correlate with positive changes for many different types of goals, such as weight management [5], chronic disease management [6], and substance use [7]. Furthermore, applying motivational interviewing in virtual environments has proven effective in encouraging behavior change [8,9].

Although such techniques can lead to positive change in participants, they may be inaccessible to people who lack access to the resources or time that the techniques require [10,11]. This problem can compound in situations where a sudden event such as the COVID-19 pandemic overwhelms health care resources and disproportionately deprives vulnerable subpopulations of health care resources [12]. Recent studies have proposed automated systems to grant more patients access to therapeutic techniques [13,14]. However, current dialogue systems often use generic or irrelevant prompts that do not adapt to participants’ responses, which may result in less engagement and possibly less behavior change from patients [15]. To address this shortcoming, we evaluated a system that integrates aspects of expressive writing and motivation interviewing into an interactive dialogue agent that adapts to participant writing behavior.

In this study, we extended our previously developed system, Expressive Interviewing [16], to engage users to reflect on the pandemic, with the goal of reducing stress and encouraging positive behavior change. We recruited 151 participants through an online survey to test the effect of Expressive Interviewing on a variety of psychosocial and behavioral outcomes. Our target population for the study was people who were open to trying new forms of technology and who, during the early stages of the pandemic, had concerns about COVID-19 that they wanted to share in writing.

We investigated the following research questions (RQs) with respect to Expressive Interviewing:

1. RQ1: What are the short-term effects of Expressive Interviewing on an individual’s mental health?
2. RQ2: What are the long-term effects (after 2 weeks) of Expressive Interviewing on an individual’s behavior?
3. RQ3: How do the short-term and long-term effects vary based on Expressive Interviewing participants’ writing style and demographic characteristics?

Our study addressed these questions through quantitative analyses of the survey responses from task and control participants before and after the intervention.

To identify short-term and long-term effects of the intervention, we focused on the following dependent variables as possible areas of change: stress, COVID-19–related mental health, COVID-19 awareness, social gathering, social discussions, and general mental health. The mental health factors were chosen to align with previous research showing how automated dialogue agents can provide effective support for mental health concerns [15]. The social behavior factors and COVID-19–specific health factors were chosen to study the possible uncertainty around norms and rules that most US residents were facing at the time of the study (ie, mid-vaccine rollout). Considering the prior work that investigates the impact of chatbots on COVID-19–related issues such as vaccination [17], we studied health outcomes that had a clear connection to both individual-level behaviors (eg, vaccination) and collective behaviors (eg, socialization).

## Methods

### Expressive Interviewing System

**Figure 1:**
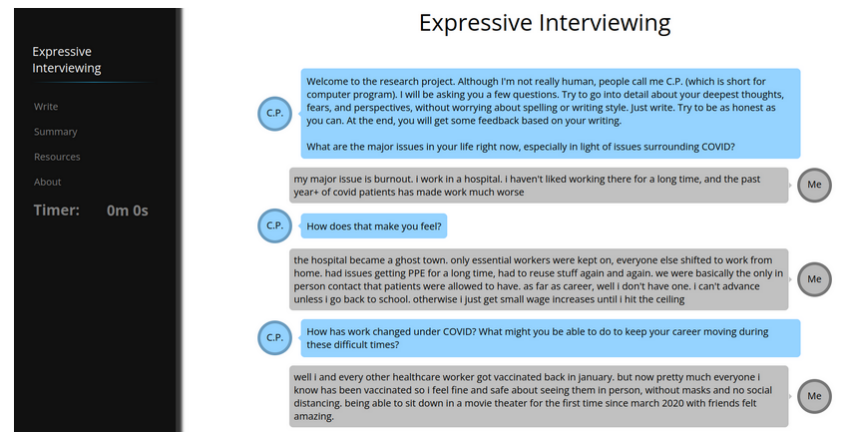
Example interaction with the Expressive Interviewing agent using sample input from a study participant. CP: computer program.

The Expressive Interviewing system was built on principles of expressive writing and motivational interviewing, drawing from the values of both writing about personal experiences and empathetic communication through reflective listening [18,19]. A previous version of the system was described by Welch et al [16]. We describe the system’s general functions in the following paragraphs and show an example interaction in Figure 1.

The system conducts an interview-style interaction with users about how the COVID-19 pandemic has been affecting them. The system’s goal is to encourage users to write as much as possible to explore their thoughts and feelings surrounding features of COVID-19. The interview consists of a set of writing prompts in the form of questions about specific issues related to the pandemic. This follows from prior research in expressive writing that shows the cathartic benefits of guided writing [4]. The system guides the interaction based on users’ responses, provides reflective feedback, and asks additional questions whenever appropriate. Although the system has a chat-like interface, participants are asked to write long responses, in contrast with a more open-domain chat setting without strong expectations for the human participant.

In order to provide reflective feedback, the system automatically detects the topics being discussed (eg, work, family) or emotions being expressed (eg, anger, anxiety) and responds with reflections that ask participants to further expand upon their feelings, ask what they can do to help improve a situation for themselves or others, or ask how one can best cope with their feelings. These reflections are direct responses to the feelings or topics mentioned, making them prompts that ask the user to write more about the situation or what they can do to change it. For instance, if the system detects *work* as a topic of interest, it responds with “How has work changed under COVID? What might you be able to do to keep your career moving during these difficult times?” Empathy is expressed by showing an understanding of what the participant is saying. Reflections contain phrases acknowledging the participant’s emotion (eg, “There is sadness in your writing”) or the subject of concern (eg, “You mention issues related to money and finance”). After the interaction ends (ie, all prompts have been answered by the user), the system provides detailed visual and textual feedback. Reflection can express empathy and is often perceived as affirming.

Each conversation consists of a series of 4 main writing prompt questions. The prompts were iteratively designed in collaboration with experts in psychology, health communications, and public health with specialties in expressive writing and motivational interviewing. The prompts for the Expressive Interviewing system are described in the list that follows. The order of the latter 3 is not fixed (eg, some people saw the “looking forward” prompt after the “advice” prompt). Note that some prompts have undergone wording changes as compared with Welch et al [16] to reflect the ongoing state of the pandemic.

- What are the major issues in your life right now, especially in the light of issues surrounding COVID-19?
- What is something you look forward to doing in the upcoming year?
- What advice would you give other people about how to cope with any of the issues you are facing?
- COVID-19 continues to affect our lives in many ways, but people have the amazing ability to find good things even in the most challenging situations. What is something that you have done or experienced recently that you are grateful for?

**Figure 2:**
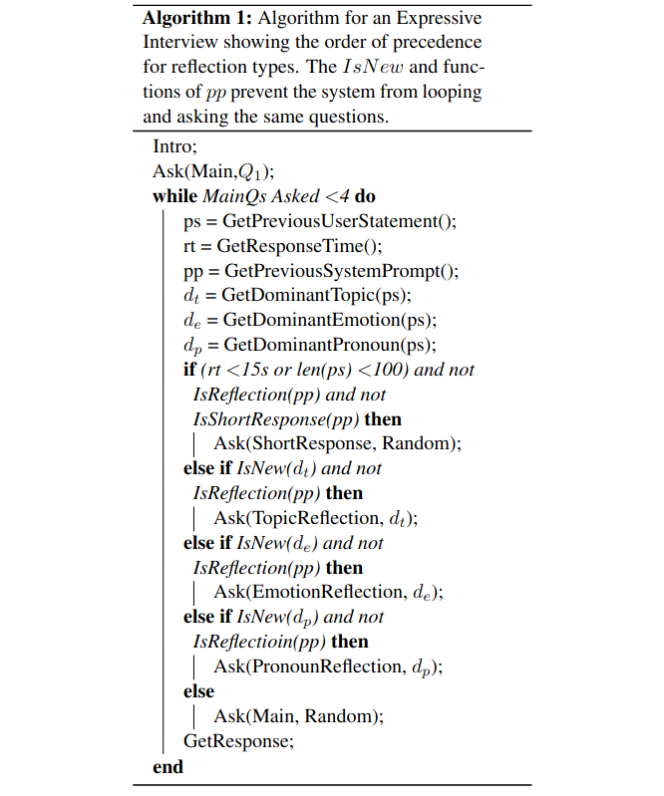
Summary of the Expressive Interviewing algorithm for choosing prompts, based on participant behavior (reproduced from Welch et al [16] which is published under Creative Commons Attribution 4.0 International License [21]).

The system relies on rules rather than machine learning, which is a design choice motivated by the risks of deploying generative models, especially in regard to sensitive topics and mental health issues [20]. This also controls the dialog in such a way that participants have somewhat similar experiences, answering at least 4 of the same prompts (per topic). The full algorithm is provided in Figure 2.

After the interaction, users are shown graphical and templated textual feedback describing their word usage: how meaningful, how self-reflective, and how their emotional tone sounded. The system is hosted on a server belonging to our research lab and was easily accessible online during the study.

### Experimental Design

To evaluate the system, we recruited 2 groups of participants from the crowdsource platform Prolific, and we randomly assigned them to the Expressive Interviewing and control (no Expressive Interviewing) conditions. All participants were prevented from participating in more than one condition to better isolate the effects of the Expressive Interviewing task. The participants were not told beforehand into which condition they would be placed, to reduce the chance of selection bias. The task participants and control participants completed the same presurvey, which included questions about the participant’s background as well as their behaviors relating to COVID-19 safety (eg, socializing in public) and general mental health (depression prevalence). The task participants were then immediately redirected to the Expressive Interviewing website afterward to begin their session. The task participants completed another short survey immediately before and after the task about their mental state, to address short-term effects.

Note that the control participants only completed a survey to track the long-term outcome variables (ie, no tracking of short-term outcome variables), unlike the Expressive Interviewing participants who completed surveys for both long-term and short-term outcome variables.

At 2 weeks after the initial presurvey, we sent an identical postsurvey to all participants in the Expressive Interviewing and control groups. The control participants were less likely to finish the experiment than the Expressive Interviewing participants, but all conditions had reasonable completion rates (63/100, 63% control; 88/100, 88% Expressive Interviewing).

Participants were compensated for their time in the study with a payment of US $4.50 for the pre-intervention survey and postintervention survey, under an assumption of 30 minutes per task and US $9 per hour as a reasonable wage. Participants in the Expressive Interviewing condition received a bonus payment of US $1.00 for completing the surveys and the treatment. We reminded participants that they needed to spend at least 15 minutes on their writing task and that their payment would be rejected if the system logged less than 15 minutes of writing.

We show a summary of the participant data, including demographics, in Table 1.

**Table 1:** Participant aggregate statistics.

Participant statistics			Expressive Interviewing (n=88)		Control (n=63)
**Education, n (%)**					
	Associate degree	10 (11)		6 (10)	
	Bachelor’s degree	29 (33)		20 (32)	
	High school	24 (27)		23 (37)	
	Master’s degree	22 (25)		15 (24)	
	PhD or higher	2 (2)		2 (3)	
	Prefer not to say	1 (1)		1 (2)	
	Some high school	1 (1)		1 (2)	
**Ethnicity, n (%)**					
	Asian or Asian American	11 (13)		7 (11)	
	Black or African American	14 (16)		6 (10)	
	Latinx or Hispanic	4 (5)		2 (3)	
	Other	2 (2)		2 (3)	
	White or Caucasian	58 (66)		51 (81)	
**Gender, n (%)**					
	Female	36 (41)		37 (59)	
	Male	48 (55)		26 (41)	
	Nonbinary	3 (3)		4 (6)	
	Other	2 (2)		1 (2)	
Age (years), median			32		29.5
**Interview**					
	Response length (words), mean	53.3		N/A^a^	
	Interview time (minutes), mean	15.2		N/A	

^a^N/A: not applicable.

### Ethical Considerations

This study was approved by the institutional review board at the University of Michigan (HUM00182586). Informed consent was obtained from all individual participants included in the study. We provided participants with the choice to opt out of the study when they opened the pre-intervention and postintervention survey. We provided the following guarantee of anonymity to participants before they begin interacting with the Expressive Interviewing system: “Everything you write will be kept completely confidential. In fact, since the interview is conducted by a computer program, you should feel free to be even more honest and direct than you might usually be. Note that this is meant to be a personal interview to learn more about your reactions to the pandemic. Hopefully, by answering these questions, you will learn more about your own reactions to the outbreak.” We did not ask for personal identifiers and data are stored anonymously. The Expressive Interviewing system and participant response data were stored on a secure server and were only accessed by the study authors. The website is secured with SSL following recent security recommendations. The conversation data cannot be de-anonymized due to the personal nature of the conversations and so will not be made publicly available.

### Example System Output

In Figure 1, we show an example interaction of a study participant with the system. We highlight the adaptive nature of the Expressive Interviewing system: In response to the participant’s discussion of work conditions, the system asks a question about the effect of the pandemic on work in general (“How has work changed under COVID?”).

## Results

### Aggregate Outcomes

#### Short-term Outcome Variables

Our study investigated the following short-term variables, which task participants were asked to provide immediately before or after completing Expressive Interviewing (all values scaled from 1 to 7):

- Life satisfaction (before): In general, how satisfied are you with your life?
- Stress (before/after): How stressed are you feeling right now?
- Meaningful (after): How valuable and meaningful was the interview to you?
- Personal (after): Overall, how personal was the interview to you?

We show the distribution of all the short-term outcome variables in Table 2. Although most participants tended to report feeling highly satisfied with their life and happy with the experience, the Expressive Interviewing participants showed a significant decrease in reported stress after completing the task (absolute change –0.80, ~23% of the original value; see Table 3).

**Table 2:** Expressive Interviewing participant short-term outcome responses (Expressive Interviewing task participants only).

Outcome variable	Description	Results, mean (SD)
Pretask stress	How stressed are you feeling right now?	3.41 (1.82)
Posttask stress	How stressed are you feeling right now?	2.58 (1.57)
Meaningful	How valuable and meaningful was the interview to you?	5.32 (1.76)
Personal	Overall, how personal was the interview to you?	5.58 (1.58)
Life satisfaction	In general, how satisfied are you with your life?	4.72 (1.58)

**Table 3:** 1-way ANOVA test of difference for changes in short-term outcome variables (Expressive Interviewing task participants only).

Outcome variables	Difference	*F* (df)	*P* value
Stress	–0.80	19.48 (87)	<.001

#### Long-term Outcome Variables.

We aggregated the individual long-term outcome variables in our analysis (using a simple mean to aggregate multiple variables), based on whether they measure similar constructs (eg, different aspects of social behavior include going out to eat and meeting friends in public). The aggregated variables are explained in Table 4, and the individual variables that are combined to form each group variable are also listed. The scale of each aggregate variable is 0 to 7 (number of days per week spent on a behavior).

**Table 4:** Summary of aggregate long-term outcome variables.

Outcome variable	Subcomponent variables^a^	Results, mean (SD)
COVID-19 mental behavior	COVID-control, COVID-hope, COVID-plan, COVID-sleep, COVID-worry (5)	4.42 (1.24)
COVID-19 awareness	COVID-recommend, COVID-talk, COVID-nervous-others, COVID-reading, COVID-watching (5)	2.27 (1.79)
Social activity	Social-face-to-face, Social-gathering, Social-public, Social-restaurant (4)	1.74 (1.29)
Social discussions	Discussions-digital, Discussions-message (2)	4.02 (1.61)
Mental health	Mental-changes, Mental-depressed, Mental-interest, Mental-nervous, Mental-worry (5)	0.85 (0.77)

^a^See Multimedia Appendix 1 for explanations of the subcomponent variables.

To explain the long-term dependent variables, we provide the following example. The “COVID-control” subcomponent variable (corresponding to the COVID-19 mental behavior outcome) represents the participants’ responses to the question “In the last week, on how many days did you feel in control of your life and able to handle challenges that might come your way?” The participants could respond with a number between 0 and 7 to indicate the duration (in days) of the feeling of being in control and able to handle challenges.

We compared the long-term changes in the outcome variables from pre to posttask (after a 2-week gap) against the changes for the control participants. We performed an ANOVA for differences between the presurvey and postsurvey conditions (see Table 5). We found a significant difference in COVID-19 awareness postsurvey compared with presurvey (mean difference=–0.267; *F*_1_=21.7, *P*<.001) and a significant difference in social activity between the Expressive Interviewing and control groups (mean difference=0.151; *F*_1_=4.75, *P*=.03). The decrease in COVID-19 awareness may be related to the decreasing importance of COVID-19–related protocols at the time of the survey (May 2021 and June 2021).

**Table 5:** 2-way ANOVA tests for effect of the experiment condition (Expressive Interviewing vs control) and survey time (pre-experiment vs postexperiment) on all outcome variables.

Condition			Sum of squares		*F* (df)		*P* value
‍**COVID-19 mental behavior**							
	Experiment condition	1.227		0.807 (1)		.37	
	Survey time	0.262		0.172 (1)		.68	
	Experiment condition * survey time^a^	0.677		0.446 (3)		.505	
	Residual	743.131		N/A^b^		N/A	
‍**COVID-19 awareness**							
	Experiment condition	8.791		3.165 (1)		.08	
	Survey time	60.380		21.742 (1)		<.001	
	Experiment condition * survey time	0.042		0.015 (3)		.90	
	Residual	1360.800		N/A		N/A	
‍**Vaccination**							
	Experiment condition	1.190		1.442 (1)		.23	
	Survey time	2.552		3.091 (1)		.08	
	Experiment condition * survey time	0.087		0.105 (3)		.75	
	Residual	403.628		N/A		N/A	
‍**Mental health**							
	Experiment condition	0.184		0.291 (1)		.59	
	Survey time	1.234		1.954 (1)		.16	
	Experiment condition * survey time	0.267		0.422 (3)		.52	
	Residual	308.861		N/A		N/A	
‍**Social activity**							
	Experiment condition	7.722		4.750 (1)		.03	
	Survey time	2.294		1.411 (1)		.24	
	Experiment condition * survey time	0.066		0.040 (3)		.84	
	Residual	794.938		N/A		N/A	
‍**Social discussions**							
	Experiment condition	0.008		0.003 (1)		.96	
	Survey time	4.000		1.624 (1)		.20	
	Experiment condition * survey time	0.002		0.001 (3)		.98	
	Residual	1204.258		N/A		N/A	

^a^The asterisk indicates interaction between different independent variables.

^b^N/A: not applicable.

### Between-Group Comparisons

Some of the outcome variables of this study, such as mental health, are known to correlate with demographics [23], and it is also plausible that some participants’ backgrounds might have influenced their interaction with the Expressive Interviewing system (eg, prior vaccination status). Considering this, we therefore investigated whether the outcomes vary at all based on participant demographic or subgroup. We ran a separate ANOVA for each participant subgroup category, including gender, ethnicity, education level, age, and vaccination status (no vaccination, partial vaccination, and full vaccination).

To compare subgroups between conditions, we used the following formula to fit the ANOVA model: Outcome variable ~ Participant subgroup + Survey time + Survey condition + Participant subgroup * Survey condition + Participant subgroup * Survey time

For brevity, we only report significant effects for the interaction between participant subgroup and survey condition (eg, whether male participants in the Expressive Interviewing condition had a different outcome than female participants in the Expressive Interviewing condition). We did not report significant effects for the participant subgroups on their own (eg, whether all male participants in the Expressive Interviewing condition and the control condition experienced a change in outcome).

We found significant effects for participant subgroups on outcome variables, after correcting for multiple comparisons: *P*=.05/(5 participant subgroups * 7 outcome variables)=0.00143. See Table 6.

For the one significant interaction effect that we did find (social activity outcome ~ ethnicity * condition), the differences between subgroups were as follows. In the Expressive Interviewing condition, African American participants had a higher rate of social activity than participants of a different ethnicity (Caucasian: *U*=4490, *P*<.001; Asian American: *U*=596; *P*<.001). In the control condition, Asian American participants had a higher rate of social activity than African American participants (*U*=114, *P*=.045), Caucasian participants (*U*=373, *P*=.004), and Hispanic participants (*U*=47.5, *P*=.04).

Most of the differences are not relevant for our study, because there were differences between the aforementioned subgroups but not between conditions (ie, not between Expressive Interviewing and control groups). For example, male participants did not show a significant difference in COVID-19 mental behavior between the Expressive Interviewing and control conditions, even though we found a significant difference based on gender for COVID-19 mental behavior in general.

We report all median values for the outcome variables for the aforementioned subgroups in Table 7.

**Table 6:** Effects for participant subgroups on outcome variables.

Outcome variables and participant subgroups		*F* (df)	*P* value
**COVID-19 mental behavior**			
	Gender	17.9 (1)	<.001
**COVID-19 awareness**			
	Gender	15.5 (1)	<.001
**Vaccination**			
	Ethnicity	15.5 (4)	<.001
	Education	7.06 (6)	<.001
	Gender	11.3 (1)	<.001
**Mental health**			
	Age	14.8 (2)	<.001
	Education	8.75 (6)	<.001
	Gender	23.8 (1)	<.001
**Social activity**			
	Ethnicity	6.18 (4)	<.001
	Ethnicity * condition	5.93 (9)	<.001
	Education	7.10 (6)	<.001
	Vaccination	12.7 (2)	<.001
**Social** **discussions**			
	Education	12.2 (6)	<.001
**Stress**			
	Age	24.4 (2)	<.001

**Table 7:** Comparison of the social activity outcome variable between participants based no ethnicity.

Ethnicity	Control, median	Expressive Interviewing, median
African American	1.5	2.5
Asian American	1	2.375
Caucasian	1.125	1.5
Hispanic	1	1.375
Other	0.5	1.25

### Correlation With Writing Behavior

Having analyzed the aggregate and subgroup trends in outcome variables, we next analyzed the participants’ writing behavior within the Expressive Interviewing condition. We focused on the correlations with both short-term effects and long-term effects. To quantify linguistic patterns, we leveraged several word categories from Linguistic Inquiry and Word Count (LIWC) [24] to characterize participant behavior through their interview responses (eg, the 1-word category contains “anxiety”-related words such as “nervous,” which we find highly useful in understanding participant responses to COVID-19–related problems).

#### Short-term Effects

As described in the previous sections, participants demonstrated a consistent decrease in self-reported stress immediately after finishing the task (see Table 3). We then investigated whether that decrease is correlated with particular language patterns. We correlated all participants’ interview language variables with their change in self-reported stress, presurvey life satisfaction, and postsurvey rating of the task as meaningful and personal. Some of these interview variables showed possible *outlier* effects (eg, 1 participant used more than twice as many ANGER words as the next-highest participant). We tried to limit these outlier effects by first removing all participants with interview response variables above the 95th percentile, then computing the correlation for each response variable on the filtered data.

We list all significant correlations in Table 8. A few findings are highlighted here. Using more anxiety words and fewer COVID-19 words was correlated with a decrease in reported stress. This point is particularly pertinent in the context of COVID-19 because people have reported feeling higher levels of anxiety due to social isolation [25]. Using more COVID-19 words and more positive emotion words was correlated with a more personal and more meaningful experience. Participants who reported higher life satisfaction before the session also used fewer anxiety words and fewer sad words in their responses.

**Table 8:** Univariate correlations between interview variables and short-term response variables.

Response variable	Outcome variable	*r*	*P* value
FEAR	Pretask stress	0.216	.003
POSITIVE EMOTION	Pretask stress	–0.204	.005
ANX^a^	Stress change	–0.264	.006
COVID^b^	Meaningful	0.259	<.001
POSITIVE EMOTION	Meaningful	0.243	.001
COVID^a^	Personal	0.214	.003
JOY	Personal	0.242	.001
POSITIVE EMOTION	Personal	0.242	.001
Prompt response overlap	Personal	0.196	.007
ANX	Life satisfaction	–0.210	.004
SAD	Life satisfaction	–0.207	.004

^a^Anxiety words.

^b^The “COVID” variable includes words related to COVID-19, such as “coronavirus.”

To better understand the connection between anxiety and stress, we investigated a few sample interview messages that contained high rates of anxiety words written by participants who reported a decrease in stress after the session. Some people discussed current events and their anxious feelings around them (eg, “This is just so *scary* when I see on the news that people are targeting Asians.”). Other participants mentioned general mental health struggles (eg, “I’m *worried* about becoming destitute, lonely, and depressed as I grow older and elder members of my family die.”). However, a considerable number of participants also framed anxiety in a positive light: In response to a question about advice for others dealing with COVID-19–related problems, a participant wrote “Learn a new skill, do something proactive, even volunteering would help cope with *stress* and everyday *struggles*.”

#### Long-term Effects

To address the possible effects of the interview itself, we compared the changes in outcome variables to the language choices by the task participants. We computed the Pearson correlation between the interview variables and the long-term variables. We found the following significant correlations (reported in Table 9): (1) Task participants who used a diverse vocabulary had an increase in COVID-19 awareness, (2) task participants who used a high amount of negative emotion words had an increase in mental health (ie, worse negative health), and (3) task participants who wrote longer responses and had a less diverse vocabulary also had a decrease in social activity. 

**Table 9:** Univariate correlations between interview variables and changes in long-term outcome variables.

Response variable	Outcome variable	*r*	*P* value
Lexical diversity	COVID-19 awareness	0.226	.003
NEGATIVE EMOTION	Mental health	0.229	.002
Length	Social activity	–0.276	<.001
Lexical diversity	Social activity	0.266	<.001

## Discussion

### Principal Findings

This study investigated the potential psychosocial and behavioral impact of Expressive Interviewing in the context of the COVID-19 pandemic. We investigated short-term patterns such as stress (RQ1), for long-term patterns such as social behavior (RQ2), and for between-participant patterns that included demographics and writing style (RQ3).

Our study shows that Expressive Interviewing participants (ie, treatment only) generally exhibited a short-term decrease in stress after the task. This correlation was particularly strong for participants who expressed more anxiety during their interaction with the system. As compared with the control condition, the task participants in general did not show significant long-term changes in COVID-19–related behavior, but some subpopulations of the participants did show changes regardless of condition. We found a slight difference in social activity between the Expressive Interviewing and control participants.

Our study suggests that Expressive Interviewing may help participants handle short-term stress related to COVID-19 and that people who write differently via the system may experience different long-term outcomes.

We expected some amount of change among all participants, considering that the Expressive Interviewing prompts ask users to reflect on their own mental behavior and their prospects for the future. In general, the participants who received Expressive Interviewing as “treatment” did not experience a consistent change in long-term COVID-19–related behavior or mental outcomes. However, we also saw a surprising amount of variance in results among task participants based on the *content* of their responses (eg, participants who wrote with more anxiety words also experienced a greater decrease in stress).

We interpret the findings as a useful example of the intended scope of chat-based therapy. As prior work suggests [13], we should not expect automated dialogue agents to address serious mental health problems but instead consider that they may be best suited for surface-level issues. A short-term decrease in stress is certainly nothing to dismiss, but such a change should not be considered a “fix” for a deep-seated problem such as social anxiety with respect to COVID-19–related policies [2]. Furthermore, the variation in responses based on writing style corroborates other studies with automated dialogue agents (eg, different levels of dropout based on perceived self-efficacy in therapy with a chatbot [15]). We should anticipate that people will react very differently to the same dialogue system given how disparately the pandemic has impacted different subpopulations [26].

### Limitations

The findings of this study should be taken in the context of the time in which the study was conducted. In June 2021, the United States had opened COVID-19 vaccinations to most of the adult population, and many previous restrictions about public gatherings were being lifted. This time period marked a significant change in the popular perception of the pandemic from an unbeatable disease to a problem that seemed to be controllable through vaccination [27]. The participants in our study may have considered COVID-19 to be a “solved” problem and may not have experienced a long-term reaction to the task in the same way that they would have in the earlier stage of the pandemic. Some of the variables studied should also be considered in the context of the time, such as vaccination status. Although, by June 2021, COVID-19 vaccinations were technically available to much of the public, many groups still faced difficulty or expressed skepticism at the technology due to its speed of development and the unknown efficacy in the long term [28]. Therefore, unvaccinated individuals in our study may have had multiple reasons for behaving differently after the task, which are hard to assess without further interviewing participants.

We also note that the participants recruited through Prolific may not be identical to the general population [29], raising the possibility of selection bias. In particular, crowd workers may have different values and attitudes than the general population [30], as well as different demographics due to different socioeconomic statuses among minority populations [23]. The constraints of the task may have also biased the participants’ responses, especially the requirement for a minimum amount of time spent on each response during the Expressive Interviewing task (for example, time constraints among crowd workers [22]). We do not claim that our results will generalize to the general public but instead view this study as an initial inquiry into the value of automated dialogue systems for helping with self-reflection through guided writing [13].

The political situation should also be emphasized. By June 2021, the issue of COVID-19 in the United States had become highly polarized [31], to the point where people were unlikely to be persuaded to change their behavior. This could explain the divergent responses in long-term outcomes among different subpopulations of participants (eg, increase in social activity among no-vaccine participants, who by this point had already made up their mind about their own COVID-19–related behavior). Polarization in COVID-19 attitudes may explain the importance of the interview variables (eg, anger words) in explaining differences in long-term outcomes. Participants with strong attitudes toward COVID-19 may have used Expressive Interviewing to better explore those attitudes and therefore experienced a stronger long-term change, indicated by the fact that participants who used many negative emotion words also experienced an increase in COVID-19 mental health outcomes. Further work in this direction should consider political ideology as a possible social dimension that can affect responses to Expressive Interviewing, as prior work has found that political ideology can affect responses to behavior change interventions [32].

### Conclusion

Expressive Interviewing can help people struggling with difficult life situations to navigate their mental and social health. This study focused primarily on addressing health behavior change with respect to COVID-19, but the analysis could be readily extended to other topical domains in which self-reflection could lead to behavior change. Our system provides a helpful starting point for future research, and we encourage researchers to modify the existing prompts for our study to match future social situations (eg, addressing anti-Asian hate in the wake of COVID-19 [33]). The findings of this study can also inform mental health counselors who want to use expressive writing to encourage behavior change in dynamic settings such as COVID-19. Although we found that a single intervention of Expressive Interviewing does not correspond to long-term change, future work may find that a series of interventions could improve patient outcomes by reinforcement [34]. By developing writing prompts that match the setting and the patient’s background, a counselor may be able to engage the patient more effectively.

## Appendix

Multimedia Appendix 1 Descriptive statistics about independent and dependent variables, description of independent linguistic variables, and additional regression analysis of results.

Multimedia Appendix 2 CONSORT-eHEALTH checklist (V 1.6.1).

## Abbreviations

LIWC: Linguistic Inquiry and Word Count
RQ: research question

## Data Availability

We cannot share the conversation data collected during this study due to sensitive information that participants shared during their conversations (eg, specific medical problems exacerbated by COVID-19). The de-identified survey data analyzed in the study are available from the corresponding author for research purposes only, by request.
