# Supplementary material for: Expressive Interviewing Agents to Support Health-Related Behavior Change: Randomized Controlled Study of COVID-19 Behaviors
Source: JMIR Form Res. 2023 Aug 1;7:e40277. doi: 10.2196/40277 (PMC10395645; doi:10.2196/40277)
Supplement: Multimedia Appendix 1 [file formative_v7i1e40277_app1.docx]

**Table S1.** Sample conversation between the Expressive Interviewing system and a participant (one of the authors).

| Participant | Text |
| --- | --- |
| C.P. | Welcome to the research project. Although I'm not really human, people call me C.P. (which is short for computer program). I will be asking you a few questions. Try to go into detail about your deepest thoughts, fears, and perspectives, without worrying about spelling or writing style. Just write. Try to be as honest as you can. At the end, you will get some feedback based on your writing. What are the major issues in your life right now, especially in the light of issues surrounding COVID? |
| You | We have been under lockdown for many months now. Things have been fine, but I think the lack of social in-person interaction is starting to show a little. My kids are generally happy to be just with us parents, but still they are now wondering when is the next time they will be seeing their friends. I have focused on work even more than I did before, and that has been keeping me happy. I do miss traveling though. |
| C.P. | Through all of this, your writing talks about happiness. How have you been able to maintain a positive attitude during these tough times? What would you say to others to help them have a positive outlook right now? |
| You | Keeping a positive attitude toward everything generally helps. Even the most concerning things in life can be turned around to show a positive side. I also feel this is the perfect time to revise one's habits, learn new things, grow stronger relations with immediate family - and those are also things that help one stay positive. |
| C.P. | Thank you, I would like to now ask you another question. COVID continues to affect our lives in many ways, but people have the amazing ability to find good things even in the most challenging situations. What is something that you have done or experienced recently that you are grateful for? Write at least 2-3 sentences. |
| You | The lockdown has been the biggest impact. We have also changed habits in my family with respect to grocery shopping (all is online), learning for my children -- we have a new routine for that. The part that I find the most disruptive is the inability to make plans for the future. By this time in previous years we would have made our summer plans. This year is different, we have no idea if we can travel to see family in Europe, and this has affected our life. |
| C.P. | I see. Tell me about a time when things were different. |
| You | Well, in all the years before, we always traveled during summer to see my parents and my husband's family. We really cherish this time of year, and so do my kids -- it's the only time when they get to spend time with grandparents, aunts, cousins, etc. It would be sad if we wouldn't get to see them this year... |
| C.P. | Thanks for sharing. Now let’s move on to the next question. What advice would you give other people about how to cope with any of the issues you are facing? Write at least 2-3 sentences. |
| You | Meeting people online is an as-good-as-it-gets substitute for face-to-face interactions. Meeting family and friends consistently can be of great help. This writing exercise can be of great help too... |
| C.P. | How has work changed under COVID? What might you be able to do to keep your career moving during these difficult times? |
| You | Just thinking less of things that are of concern can be helpful. But I have to say work has not been a concern. On the contrary, it's been a relief to be able to work from home. |
| C.P. | I appreciate you sharing that. Let's go in a slightly different direction. What is something you look forward to doing in the upcoming year? Please spend some time writing about this question. The more you write, the better I can understand your perspective. |
| You | Traveling! I miss seeing new places. I also look forward to seeing friends and collaborators 'in person'. As for more 'local' stuff, I look forward to going to Starbucks. And going to a bookstore. |
| C.P. | Thank you for everything you shared with me today. I do not have any more questions. Before going to your feedback, is there anything else you wanted to say? |
| You | No, I enjoyed talking to you today. Thank you for listening! |

# **Outcome variables**

Here we show the descriptions and distributions of the individual outcome variables for all participants, which comprise the aggregate outcome variables such as “COVID mental behavior” referenced throughout the study (see Table 4). We provide a full summary of the individual outcome variables as follows: social activity (Table S2), COVID-related behavior (Table S3), mental health (Table S4).

**Table S2.** Summary of individual outcome variables (social factors).

| Sub-component variables | Description | Mean (SD) |
| --- | --- | --- |
| Social-face-to-face | In the last week, on how many days did you do the following in the presence of people who were not fully vaccinated or with unknown vaccination status: - Have a face-to-face meeting with someone outside your home | 1.986 (2.111) |
| Social-public | In the last week, on how many days did you do the following in the presence of people who were not fully vaccinated or with unknown vaccination status: - Go to a public place | 3.105 (2.092) |
| Social-restaurant | In the last week, on how many days did you do the following in the presence of people who were not fully vaccinated or with unknown vaccination status: - Eat at a restaurant with other people | 0.870 (1.315) |
| Social-gathering | In the last week, on how many days did you do the following in the presence of people who were not fully vaccinated or with unknown vaccination status: - Go to a gathering with more than 8 people (such as a party, religious service, sporting event) | 0.713 (1.391) |
| Discussions-digital | Estimate as well as you can on how many days (in the last week) you had one-on-one meetings (not group meetings with multiple people at the same time) on the phone, skype, zoom, facetime, or other digital medium in the last week. | 2.502 (2.202) |
| Discussions-message | Think about communications that you sent (in the last week) to just one person at time (not mass mailings, or ccs on emails) using email, text or SMS, or other digital text Discussions-message. Estimate how many days in the last week you sent these Discussions-messages. | 5.401 (2.105) |

**Table S3.** Summary of long-term outcome variables (COVID-related).

| Question ID | Description | Mean (SD) |
| --- | --- | --- |
| Public-place-mask | In the last week, when you were in public places, on how many days did you: - Wear a mask | 3.719 ± 2.605 |
| Public-place-no-mask | In the last week, when you were in public places, on how many days did you: - NOT wear a mask | 1.269 ± 2.135 |
| Vaccination (value converted to numeric: 0=no vaccine, 1=at least 1 dose, 2=fully vaccinated) | Have you been fully vaccinated? | 1.178 ± 0.922 |
| COVID-talk | In the last week, on how many days did you - Talk to people about COVID-19 and/or vaccinations | 2.052 ± 1.916 |
| COVID-test | In the last week, on how many days did you - Get tested for COVID-19 | 0.149 ± 0.619 |
| COVID-recommend | In the last week, on how many days did you - Recommend others to be tested or vaccinated for COVID-19 | 0.802 ± 1.419 |
| COVID-worry | In the last week, on how many days did you – Not have intrusive thoughts or worries about COVID-19 | 5.725 ± 1.939 |
| COVID-sleep | In the last week, on how many days did you – Not have difficulty sleeping due to worries about COVID-19 | 6.597 ± 1.228 |
| COVID-hope | In the last week, on how many days did you - Feel hopeful that things are starting to return to normal after COVID-19 | 3.333 ± 2.518 |
| COVID-plan | In the last week, on how many days did you - Make plans to do things that were "normal" before COVID (like travel) | 2.045 ± 2.090 |
| COVID-control | In the last week, on how many days did you - Feel in control of your life and able to handle challenges that might come your way | 3.915 ± 2.558 |
| COVID-reading | In the last week, on how many days did you read an article about - COVID-19 | 2.126 ± 2.098 |
| COVID-watching | In the last week, on how many days did you watch a movie, YouTube, news show, or television program about - COVID-19 | 1.407 ± 2.054 |
| COVID-worry | In the last week, on how many days did you - Worry about getting COVID | 1.066 ± 1.854 |
| COVID-nervous-others | In the last week, on how many days did you - Feel nervous around people because they might have COVID | 1.192 ± 1.839 |

**Table S4.** Summary of long-term outcome variables (mental health variables).

| Question ID | Description | Mean (SD) |
| --- | --- | --- |
| Mental-nervous | Over the last two weeks, how often have you been bothered by the following problems? - Feeling nervous, anxious or on edge | 0.983 ± 0.948 |
| Mental-worry | Over the last two weeks, how often have you been bothered by the following problems? - Not being able to stop or control worrying | 0.789 ± 0.946 |
| Mental-depressed | Over the last two weeks, how often have you been bothered by the following problems? - Feeling down, depressed or hopeless | 0.771 ± 0.936 |
| Mental-interest | Over the last two weeks, how often have you been bothered by the following problems? - Little pleasure or interest in doing things | 0.769 ± 0.917 |
| Mental-changes | Over the last two weeks, how often have you been bothered by the following problems? - Concern with changes that the future will bring | 1.209 ± 0.991 |

# **Lexicons**

In our analysis, we leverage the LIWC lexicons to understand patterns of emotion and cognition among Expressive Interviewing participants. We list a sample of the categories with their example words and sentences in Table S5.

**Table S5.** LIWC lexicon example words and matching responses. Full list omitted for space.

| Word category | Sample words | Sample response |
| --- | --- | --- |
| ADJECTIVE | as, more, better, new, most, than, best, happy, normal, before | I have **more** free time to spend reading to him and playing. |
| ADVERB | so, about, also, just, now, really, there, when, how, very | I consider my health to be a major issue right **now**, as well as that of my family’s. |
| AFFECT | grateful, good, better, well, love, sure, worry, best, care, happy | I will move to another country and find a **good** job. |
| ANGER | hate, angry, kill, frustrating, critical, suck, killing, spite, bother, frustrated | I work less now because of it. It **sucks**. |
| ANXIETY | worry, anxiety, fear, worried, stressful, stressed, struggling, stress, afraid, anxious | I’m too **stressed** to go out and do things. |
| COGNITIVE PROCESS | not, but, all, would, because, or, think, if, really, make | I need to find a way to **make** more money **but** also juggle three children, a busy lifestyle and **all** that. |
| DRIVES | we, family, work, get, able, up, try, our, help, good | My **family** had COVID-19 a couple of months ago and I had long haulers symptoms. |
| FEAR | fear, afraid, anxious, scared, terrible, scary, panic, unsure, horrible, dash | At first when it broke out I was really **anxious** and **afraid** for me and my family but now I think my **fear** of COVID has subsided a lot. |
| FOCUSPRESENT | have, is, be, am, are, can, do, has, work, get | I think first you need to **have** a positive view about the uncertain future and a hope that everything will **be** back to the normal. |
| INSIGHT | think, feel, know, find, feelings, believe, become, thought, seem, learn | I am always **thinking** about how I can help other people. |
| JOY | good, love, worry, happy, enjoy, great, move, close, concern, happiness | I am looking forward to this moment when I can **enjoy** my holiday like the years before COVID-19. |
| NEGATIVE EMOTION | worry, problems, difficult, anxiety, worried, fear, bad, stressful, lost, alone | **worry** that I will be unable to complete my goals and tasks for my employer. |
| POSITIVE EMOTION | grateful, good, better, well, love, sure, care, best, happy, important | I haven’t really thought of that recently but my goal has always been to be **better** than I was previously, to bring joy and happiness to those I **love** the most and focus on progressing in my career. |
| PERSONAL PRONOUN | I, my, me, you, we, they, them, your, our, their | **I** feel like **my** problems are solvable in time. |
| RELATIVE | in, on, time, at, out, forward, now, up, go, when | During this hard **time** my family had to travel **out** for safety since it was no longer safe **in** the country. |
| SAD | down, bad, sad, depression, weight, depressed, low, depressing, miserable, suffering | I feel **depressed** and lonely |
| SOCIAL | you, we, people, family, they, them, your, their, our, help | I have invited several friends and **family** members and **they** are planning on coming. |
| TIME | time, forward, now, year, when, back, during, new, always, since | We have been saving up all of our money for over a **year** **now**. |

## **Regression analysis**

In spite of the trends from single-variable analysis, we acknowledge that some participant attributes may be correlated with one another and require additional consideration. At the time of data collection (June 2021), the older participants were more likely to have received at least one dose of the COVID vaccine, simply because in the United States older people had earlier access to the vaccine than young people. We set up a regression task for each outcome variable and use ordinary least squares regression to learn parameter weights for the following sets of predictive variables. In some cases we restrict to only task participants due to limitations of the data (ie, control participants did not provide any writing data to analyze).

- Demographics (all data)
- Demographics + Interviews (task participants only)
- Interviews + Question types (task participants only)

We found the following significant effects after fitting the regressions on all participants (all effects shown in Table S6).

- Study participants with no vaccination before the study reported a decrease in Mental health, which is positive (e.g., less time spent worrying).
- When considering just interview variables, task participants who expressed more anger and less fear in their writing reported a decrease in Mental health (i.e.improved mental health). When considering interview variables and questions, task participants who expressed more anger, less fear, and more cognitive process words reported a decrease in Mental health.
- Participants with lower education, e.g., with some high school education, had sharper decreases in COVID mental behavior (e.g., less time feeling hopeful about COVID) as compared to higher education participants.
- When considering interview variables and questions, task participants who expressed less sadness, used a higher amount of personal pronouns, and expressed less positive emotion had an increase in vaccination. Expressing emotion in the Expressive Interviewing setting may prevent people from reflecting on the consequences of their own actions (i.e., use of personal pronouns), and these consequences could in turn make hesitant people ready to consider vaccination.
- When considering interview variables and questions, task participants who expressed more anger and less negative emotion, and who received questions related to anxiety or health, had an increase in social discussions. This may be a result of such questions prompting the release of anger around COVID and a subsequent desire to engage with others in real life rather than talk with a chatbot.

**Table S6.** Significant estimates from regressions on all outcome variables and predictor variable subsets.

| Predictors | Outcome | Parameter | β | Z | p |
| --- | --- | --- | --- | --- | --- |
| Demo | COVID mental behavior | Education (Some High School) | -3.495 | -3.03 | 0.003 |
| Demo | Vaccination | Vaccination (single dose) | 0.585 | 10.7 | <*0.001* |
| Demo | Mental health | Vaccination (no doses) | -0.304 | -2.60 | 0.01 |
| Demo+ Interview | COVID mental behavior | Education (Associate Degree) | -3.218 | -2.38 | 0.02 |
| Demo+ Interview | COVID mental behavior | Education (Bachelor’s Degree) | -3.533 | -2.61 | 0.01 |
| Demo+ Interview | COVID mental behavior | Education (High School) | -3.996 | -3.08 | 0.003 |
| Demo+ Interview | COVID mental behavior | Education (Master’s Degree) | -3.649 | -2.76 | 0.008 |
| Demo+ Interview | COVID mental behavior | Education (Ph.D. or higher) | -3.632 | -2.31 | 0.02 |
| Demo+ Interview | COVID mental behavior | Education (Some High School) | -6.738 | -3.64 | <*0.001* |
| Demo+ Interview | Vaccination | Vaccination (single dose) | 0.540 | 6.31 | *0.001* |
| Demo+ Interview | Mental health | Vaccination (no doses) | -0.371 | -2.55 | 0.01 |
| Demo+ Interview | Mental health | ANGER | -0.087 | -2.24 | 0.03 |
| Demo+ Interview | Mental health | FEAR | 0.110 | 2.70 | 0.009 |
| Demo+ Interview | Social activity | Gender (F) | 2.008 | 2.07 | 0.04 |
| Demo+ Interview | Social activity | Gender (M) | 2.023 | 2.10 | 0.04 |
| Interview+ Question | COVID mental behavior | NEGATIVE EMOTION | -0.619 | -2.40 | 0.02 |
| Interview+ Question | Vaccination | SAD | -0.065 | -3.06 | 0.003 |
| Interview+ Question | Vaccination | PERSONAL PRONOUN | 0.465 | 2.52 | 0.01 |
| Interview+ Question | Vaccination | POSITIVE EMOTION | -0.221 | -2.42 | 0.02 |
| Interview+ Question | Mental health | ANGER | -0.086 | -2.24 | 0.03 |
| Interview+ Question | Mental health | FEAR | 0.118 | 2.99 | 0.004 |
| Interview+ Question | Mental health | COGNITIVE PROCESS | -0.676 | -2.30 | 0.02 |

| Predictors | Outcome | Parameter | β | Z | p |
| --- | --- | --- | --- | --- | --- |
| Interview+ Question | Mental health | Question (health) | -0.414 | -2.15 | 0.03 |
| Interview+ Question | Mental health | Question (work) | -0.409 | -2.69 | 0.009 |
| Interview+ Question | Social discussions | ANGER | 0.329 | 3.24 | 0.002 |
| Interview+ Question | Social discussions | NEGATIVE EMOTION | -0.774 | -2.41 | 0.02 |
| Interview+ Question | Social discussions | Question (Generic 1) | 1.068 | 2.87 | 0.006 |
| Interview+ Question | Social discussions | Question (Generic 3) | 0.841 | 2.09 | 0.04 |
| Interview+ Question | Social discussions | Question (Generic 7) | 0.872 | 2.05 | 0.04 |
| Interview+ Question | Social discussions | Question (anxious) | 1.825 | 3.61 | < |
| Interview+ Question | Social discussions | Question (health) | 1.716 | 3.37 | 0.001 |

Taken together, these outcomes suggest that all participants who may not have the most awareness of COVID’s severity (eg, lower education and unvaccinated participants) may have had their beliefs reinforced by the study.
